# Case Report: Pancreatic and hepatic kaposiform hemangioendothelioma presenting as consumptive coagulopathy and right hepatic atrophy

**DOI:** 10.3389/fonc.2023.1097997

**Published:** 2023-05-02

**Authors:** Chengbo Ai, Tong Qiu, Jiangyuan Zhou, Chuan Wang, Jiulin Song, Siyu Pu, Shuguang Jin

**Affiliations:** Department of Pediatric Surgery, West China Hospital/West China School of Medicine, Sichuan University, Chengdu, China

**Keywords:** Kaposiform hemangioendothelioma (KHE), Kasabach-Merritt phenomenon (KMP), vascular tumor, Whipple operation, thrombocytopenia, case report

## Abstract

Kaposiform hemangioendothelioma (KHE) is a rare vascular tumor that causes progressive angiogenesis and lymphangiogenesis, which often occurs in the skin or soft tissue, with an acute onset and rapid progression. A 4-year-old girl was admitted to our hospital with a 2-year history of thrombocytopenia, combined with right hepatic atrophy and pancreatic lesion for 3 months. At the age of two, she developed purpura and thrombocytopenia was detected, after treatment with gamma globulin and corticosteroids, the platelet count normalized, but it dropped immediately at lower doses. One year after the cessation of corticosteroids therapy, the patient presented with abdominal pain and abnormal liver function and the magnetic resonance imaging (MRI) revealed right hepatic atrophy and pancreatic occupancy, but the first liver biopsy did not reveal any positive pathological results. By analyzing the clinical manifestations in conjunction with MRI and abnormal coagulation, we considered that the patient might be diagnosed as KHE with Kasabach-Merritt phenomenon, however, sirolimus treatment was ineffective and pancreatic biopsy only showed a tendency for tumors of vascular origin. Finally, we performed a Whipple operation after the right hepatic artery embolization, histological and immunohistochemical examination suggested KHE. Three months postoperatively, the patient’s liver function, pancreatic enzymes and blood clotting function gradually returned to normal. KHEs may result in significant blood loss with worsening of the coagulopathy and functional impairment, timely surgical intervention for KHE is necessary when non-invasive or minimally invasive treatment is ineffective, or the symptoms of tumor compression are obvious.

## Introduction

Kaposiform hemangioendothelioma (KHE) has locally aggressive characteristics and compressive effects and is often associated with the life-threatening Kasabach-Merritt phenomenon (KMP), which is characterized by consumptive coagulopathy and severe thrombocytopenia (1, 2). The estimated annual prevalence of KHE is 0.071 per 100,000 children (3). KHE often occurs in infants and young children, with an acute onset and rapid progression. Although it most commonly occurs in the skin or soft tissue, KHE can occur in noncutaneous locations without cutaneous signs, including bone, mediastinum, and retroperitoneum (4). Such KHEs may lack cutaneous manifestations and present with atypical symptoms, except for KMP, indicating that the diagnosis of such KHEs is more challenging, and the KMP possibly causes significant pain, secondary bleeding and compression of more vital structures (5, 6). In addition, the standard treatment regimens are lacking, and surgical excision is often a difficult option due to the high risk of morbidity and mortality for most KHE patients with KMP (1, 4, 6). Complex pancreatic and hepatic KHE is exceedingly rare, and biopsy-proven pancreatic and hepatic KHE has only been reported in the autopsy of a 9-day-old female baby (7). Therefore, we reviewed the case of a 4-year-old girl with a sirolimus-ineffective pancreatic and hepatic KHE.

## Case presentation

A 4-year-old girl was admitted to our hospital with a 2-year history of thrombocytopenia, combined with right hepatic atrophy and pancreatic lesion for 3 months.

In June 2017, she developed extensive purpura and thrombocytopenia. After completing ultrasound, blood smear, bone marrow aspiration biopsy, and gene mutations on whole exome sequencing of blood, the local hospital diagnosed her with immune thrombocytopenia. Following treatment with gamma globulin and corticosteroids (prednisone Acetate Tablets), the platelet count normalized, but it dropped immediately at lower doses. One year later, the parents of the child started to try herbal treatment because they were worried about the side effects of long-term hormone therapy and the patient did not have any other symptoms, although the platelet count was still low.

One year after the cessation of corticosteroids (July 2019), the patient presented with abdominal pain and abnormal liver function. After corticosteroids and hepatoprotective therapy (Glycyrrhizin), the patient’s symptoms subsided considerably and liver function returned to normal soon. However, magnetic resonance imaging (MRI) revealed a shrunken right liver lobe with a large patchy hyperintense signal on T2 weighted imaging; delayed heterogeneity intensified after enhancement. A hypointense signal (approximately 2.3 × 1.3 cm) on T1 weighted imaging was observed behind the of pancreatic head, with circular inhomogeneous enhancement. The right branch of the portal vein was not clearly visible with collateral vascularization, and the middle and right hepatic veins were poorly visualized. To clarify the cause of the right hepatic atrophy, liver puncture biopsy was performed, however, it did not suggest any positive pathological results (neither classical autoimmune liver disease changes, no acute liver injury, nor tumor-like changes).

**Figure 1:**
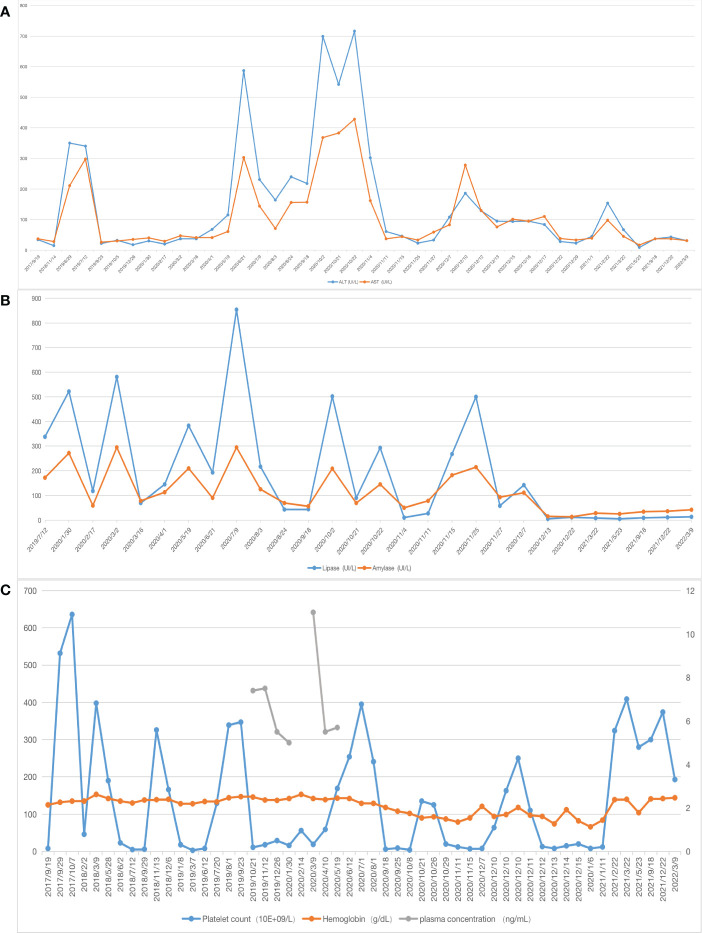
Laboratory values **(A)** X-axis represents the date, the blue line represents alanine aminotransferase (ALT),the orange one represents aspartate aminotransferase (AST); **(B)** the blue line represents lipase, the orange one represents amylase, the patient’s liver function and pancreatic enzyme levels gradually returned to normal after surgery; **(C)** the blue line represents platelet count, the orange one represents hemoglobin, the gray one represents plasma concentration, after 7 months of sirolimus treatment, the relationship between sirolimus concentration level and platelet count showed that the sirolimus treatment was ineffective.

In October 2019, the patient was referred to our hospital outpatient clinic. By analyzing the clinical manifestations in conjunction with MRI and abnormal coagulation (lowest platelet counts of 5 × 10 ^9^/L), we considered that the patient might be diagnosed as KHE with KMP. Due to the patient’s effective corticosteroids therapy and high risk of biopsy surgery, the decision was made to empirically use sirolimus after full communication with the patient’s parents. We trialed 0.08 mg/kg/day sirolimus, but the plasma concentration was maintained at only 5-5.7 ng/mL, and the platelet count remained low (**Figure 1**). In February 2020, the patient presented with recurrent abdominal pain and elevated levels of pancreatic enzymes and transaminases. CT revealed dilated intra- and extrahepatic bile ducts and pancreatic ducts. Consequently, the treatment plan was changed from sirolimus to corticosteroids administration, and platelet counts returned to normalized. Simultaneously, as stenosis was observed during endoscopic retrograde cholangiopancreatography (ERCP), stents were placed in the pancreatic and biliary ducts without any severe complications. However, the patient’s liver function and pancreatitis still showed no significant improvement, and she exhibited reduced hormone sensitivity and the platelet count decreased again (platelet count: < 20 × 10 ^9^/L).

In October 2020, we performed a fine-needle biopsy of the pancreas, however, the pathological examination showed only a characteristic for of vascular-origin tumors, and the patient developed recurrent biliary tract infections and persistent jaundice. Since prolonged medication was ineffective and the tissue biopsy failed to confirm a diagnosis, we had held several discussions with the Departments of Liver and Pancreatic Surgery, Pediatric Hematology and Pediatric Infection, and developed a surgical plan together. Additionally, the patient’s parents strongly requested surgery after discussing the patient’s condition. As the risk of performing right hepatic and pancreaticoduodenectomy with a low platelet count was very high, we initially embolized the right hepatic artery to reduce the extent of resection and risk of coagulation disorder.

**Figure 2:**
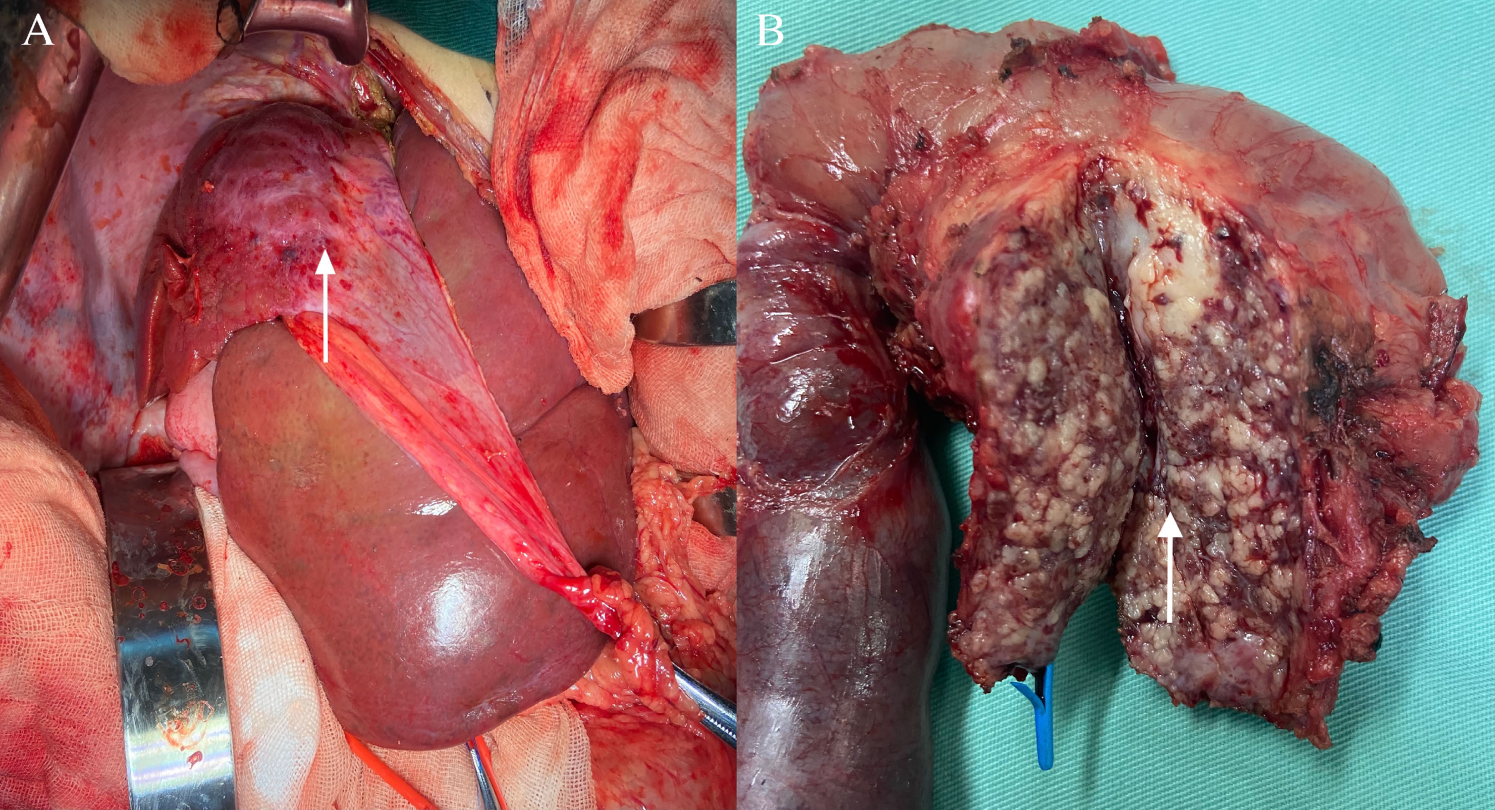
Surgical treatment. **(A)** Pancreatic kaposiform hemangioendothelioma was seen during the Whipple operation (white arrow); **(B)** Intraoperatively, the right lobe of the liver was seen to be shrunk, and liver biopsy was rereated (white arrow).

One month after embolization, the patient’s platelet count remained low (7 × 10^9^/L), and ultrasonography showed a weak echogenic mass of 4 × 2.9× 3.5 cm in the pancreatic head; therefore, we performed a Whipple operation and second liver biopsy in December 2020 (**Figure 2**).

## Result

**Figure 3:**
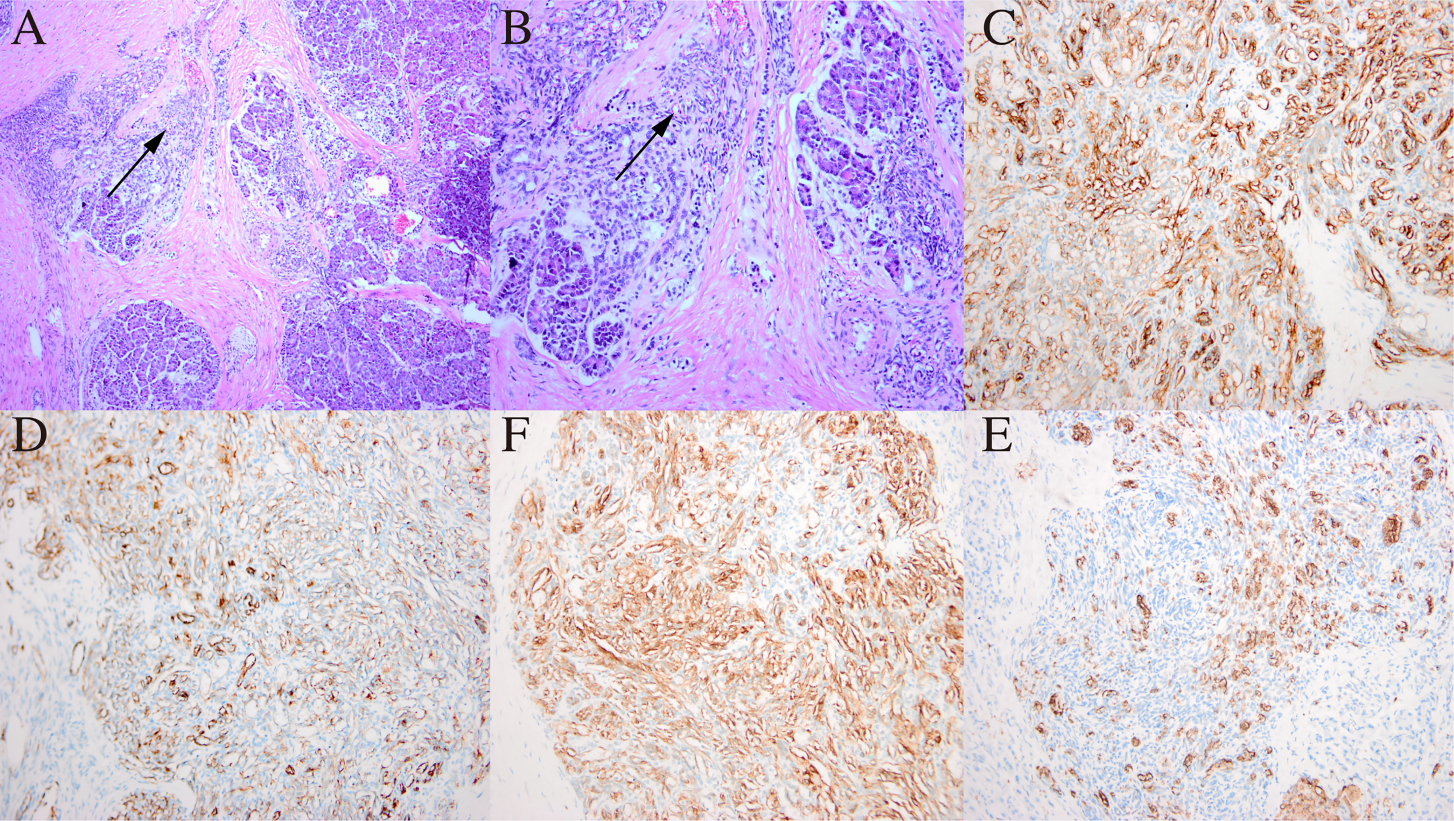
Histological examination and immunohistochemical staining of the pancreatic mass. **(A)** In HE staining, the tumor showed nestlike distribution of spindle-shaped endothelial cells (black arrow) with extensive infiltration of the pancreatic mesenchym. Original magnification: × 100. **(B)** Spindle endothelial cells aligned to form slit-like vascular lumina containing erythrocytes (black arrow). Original magnification: × 200. **(C)** CD34 positivity on immunohistochemical staining; **(D)** CD31 positivity on immunohistochemical staining; **(F)** D2-4 positivity on immunohistochemical staining; **(E)** Glut-1 negativity on immunohistochemical staining. Original magnification: × 200.

Finally, the pancreaticoduodenum and extrahepatic bile duct tumor were completely resected, with no tumor infiltration in the cutting edges of the pancreas, intestine, or biliary tract. Histological examination showed that the tumor was detected in the pancreatic, biliary and right hepatic tissues of the patient. In HE staining, the tumor showed nestlike distribution of spindle-shaped endothelial cells with extensive infiltration of the pancreatic mesenchyme, and slit-like vascular lumina containing erythrocytes (**Figures 3A, B**), which were consistent with the typical pathological manifestations of KHE (8–10). Meanwhile, there were no signs of tumor invasion were seen in the left liver and surrounding adjacent tissues, and there were no other diffuse lesions in the thoracoabdominal cavity. Immunohistochemical staining showed that endothelial cells were positive for the vascular endothelial markers CD34 (**Figure 3C**) and CD31 (**Figure 3D**) and the lymphatic endothelial marker D2–40 (**Figure 3F**), along with scattered P53 positivity and 10% Ki-67 positivity, but negative for glucose transporter-1 (**Figure 3E**) and human herpes virus-8 staining.

Postoperatively, the patient’s liver function, pancreatic enzyme levels and coagulation function gradually returned to normal, and she is still being followed up closely (**Figures 1A, B**).

## Discussion

We reviewed the literature and retrieved eight published cases of pancreatic KHE with sufficient information (**Table 1**) (11–18). Almost all cases occurred in in the pancreatic head and had KMP; presenting with biliary obstruction symptoms, such as obstructive jaundice and biliary vomiting. Combined with imaging, laboratory tests and biopsy, patients can be diagnosed clearly in time. However, the diagnosis and treatment of the patient in this study were more challenging.

**Table 1:** Reported cases of pancreatic kaposiform hemangioendothelioma.

Author	Country	Sex	Age	Chief Complaint	KMP	Platelet Count	Location	Size	Treatment	Follow-up Time	Result of Flow-up
Liang 2022	China	male	10-month-old	obstructive jaundice	No	NA	head of pancreas	3.0 × 2.8 × 2.4cm	sirolimus 0.8 mg/m2 twice daily	NA	Alive
Kim 2021	Korea	male	28-day-old	white stool and jaundice	No	NA	NA	2.1cm	pylorus-preserving pancreatoduodenectomy and interferon alpha 1month after surgery	116 months	Alive
Wei 2020	China	male	50-day-old	jaundice and cutaneous petechiae	Yes	6×10^9^/L	head of pancreas and hepatic portal area	4.9 × 3.8 × 3.5cm	Initial treatment: Steroids and VCR Sirolimus withdrawn after 1 y	12 months	Alive Normal platelet counts and liver function tumor size: Near-complete regression
Wei 2020	China	male	80-day-old	evaluation of jaundice	Yes	33×10^9^/L	head of pancreas and hepatic portal area	9.9 × 5.2 × 9.2 cm	Initial treatment: Steroids and VCR Sirolimus withdrawn after 1.5 y	32 months	Alive Normal platelet counts and liver function, tumor size: Minimal residual
Wei 2020	China	male	73-day-old	jaundice and petechiae of extremities	Yes	3×10^9^/L	head of pancreas	5.6 × 3.2 × 2.9 cm	Initial treatment: Steroids and VCR Sirolimus used for 1 y and ongoing	10 months	Alive Normal platelet counts and liver function, tumor size: Minimal residual
Mathew 2019	South Africa	female	8-month-old	obstructive jaundice	Yes	21×10^9^/L	head of pancreas	NA	interferon alpha	NA	Dead
Triana 2017	Spain	female	4-month-old	obstructive jaundice	Yes	51×10^9^/L	head of pancreas	3.3 × 2.4 ×1.8 cm	Sirolimus: 0.8 mg/m ^2^/12 h	NA	Alive
Wang 2017	China	male	16-month-old	xanthochromia with intermittent fever	No	262×10^9^/L	pancreatic head tumor attached to the descending part of duodenum	2.2 × 1.9 ×2.5 cm	Sirolimus: 0.04 mg/kg twice per day. Sirolimus concentration: 8-11 ng/ml.	6 months	Alive The size of the mass decreased.
Leung 2014	China	male	3-day	biliary vomiting	Yes	23×10^9^/L	head of pancreas	4.0 × 4.0 ×5.0cm	Whipple operation	5 months	Alive
Vogel 2006	USA	male	12-month-old	abdominal distention, anemia, and easy bruising	Yes	3×10^9^/L	pancreatic head tumor compressed the lateral duodenal wall	NA	Systemic corticosteroid, vincristine	NA	NA

KMP, Kasabach-Merritt phenomenon; NA, not available; VCR, vincristine.

Our patient initially presented with extensive purpura and thrombocytopenia, lacked skin manifestations and abdominal symptoms, and was misdiagnosed with immune thrombocytopenia. The patient had previously received extensive treatment with corticosteroids and other medications for a long time before presenting with liver atrophy; thus, a diagnosis of drug toxicity-induced liver atrophy could be easily made. Her clinical manifestations, MRI, and abnormal coagulation suggested a diagnostic direction, nevertheless, this was insufficient for a definitive diagnosis. Moreover, 7 months of sirolimus treatment was ineffective. In addition, the first liver biopsy did not puncture the diseased tissue, and the pancreatic puncture tissue specimen was too small and only indicated a vascular origin tumor. After the pancreatic puncture, the patient had severe tumor compression symptoms, including severe obstructive jaundice and biliary tract infection, even though biliary and pancreatic duct stents were placed.

Initially, we hypothesized that the primary lesion was in the liver, because the relatively larger magnitude of a liver lesion could cause more platelet depletion and that platelet retention in the hepatic veins led to right hepatic atrophy. However, the first liver biopsy revealed no positive pathological results, and the patient’s platelet count remained low after right hepatic artery embolization. Moreover, biopsy-proven hepatic KHE is even rarer than other KHEs, with only one published autopsy in a 9-day-old neonate and one bile duct case (3, 7, 19). In contrast, three cases described a pancreatic mass close to the hepatic portal area on MRI or CT scan. Yao et al. reported that obstructive jaundice was more severe in cases involving both the pancreatic head and hepatic portal area than in cases where only the head of the pancreas was involved (11, 16), yet there was no pathological confirmation, obstruction of the hepatic vessels obstruction, or right hepatic atrophy. Therefore, although it is rare, the pancreas may be the primary lesion involving the liver, with concomitant KMP resulting in platelets being trapped by abnormal blood vessels, leading to right hepatic atrophy. Postoperative pathology suggested that multiple sites of the liver, bile duct, and pancreas were involved implying that the invasive capacity of KHE may be stronger than previously considered, as KHE is considered to be an intermediate tumor type with local invasive ability (1, 8).

Due to the rarity and heterogeneity of KHE, current treatment regimens mostly lack a strong evidence base, with no clear guidelines for specific treatment. Combination therapy regimens are mostly recommended for patients with KMP (4, 6). Our patient was treated with corticosteroids, which temporarily normalized the platelet count, however, they quickly relapsed upon discontinuation or reduction and the patient tolerated corticosteroids at a later stage. In recent years, sirolimus reportedly had a clear effect on KHE with KMP and caused rare side effects (4, 20, 21). Moreover, sirolimus treatment was also effective in some cases of pancreatic KHE, but the time needed to reach the desired drug plasma levels or to improve symptoms, such as KMP and jaundice, was prolonged (11, 13). In contrast, after 7 months of sirolimus treatment, our patient’s platelet count did not improve (**Figure 1C**), and the symptoms of recurrent abdominal pain, obstructive jaundice, and pancreatitis increased. Yao et al. hypothesized that the delayed action of sirolimus may be related to biliary obstruction (11), but our patient only developed symptoms of biliary obstruction in the later stages of sirolimus use, and the bilirubin levels were normal in the pre-drug period. Therefore, we believe this may be linked to the patient’s inability to achieve the ideal drug concentration for a prolonged time, allowing further progression of the disease.

Although surgical resection is curative, non-invasive or minimally invasive treatment should be prioritized because extensive surgery for lesion infiltration into vital structures may result in significant blood loss with worsening of the coagulopathy and functional impairment (1, 6). However, treatments such as pharmacotherapy and arterial embolization were ineffective here, and tumor compression was obvious. Failure to perform timely surgical intervention may result in lost treatment opportunities.

## Conclusion

Pancreatic and hepatic KHE is rare and may only display symptoms of KMP, so when the patient has multipole organ lesions and abnormal coagulation functions are observed, KHE should be suspected. Non-invasive or minimally invasive treatments should be prioritized, however, if they are ineffective or the tumor compression and obstruction symptoms are obvious, the patient’s condition should be evaluated, and timely surgery should be performed to confirm the diagnosis and treat.

## Data availability statement

The original contributions presented in the study are included in the article/**Supplementary Material**. Further inquiries can be directed to the corresponding author.

## Ethics statement

Written informed consent was obtained from the individual(s), and minor(s)’ legal guardian/next of kin, for the publication of any potentially identifiable images or data included in this article.

## Author contributions

CA performed the database search, reviewed the literature, and drafted the manuscript. TQ reviewed the literature and drafted the manuscript. JZ, CW, JS, and SP helped draft the manuscript and performed the data extraction process. SJ was responsible for the revision of the manuscript for important intellectual content. All authors contributed to the article and approved the submitted version.

## Supplementary material

Supplementary Figure 1 MRI findings Axial T2 **(A)** MRI of the abdomen demonstrates that the right liver lobe was shrunken with a large patchy hyperintense signal, and coronal T1 **(B)** contrast enhanced MRI of the abdomen demonstrates delayed inhomogeneous intensified of the right liver lobe after enhancement. A hypointense signal was observed behind the of pancreatic head, with circular inhomogeneous enhancement.
