# Supplementary figures and images for: Case Report: Pancreatic and hepatic kaposiform hemangioendothelioma presenting as consumptive coagulopathy and right hepatic atrophy
Source: Front Oncol. 2023 May 2;13:1097997. doi: 10.3389/fonc.2023.1097997 (PMC10187787; doi:10.3389/fonc.2023.1097997)

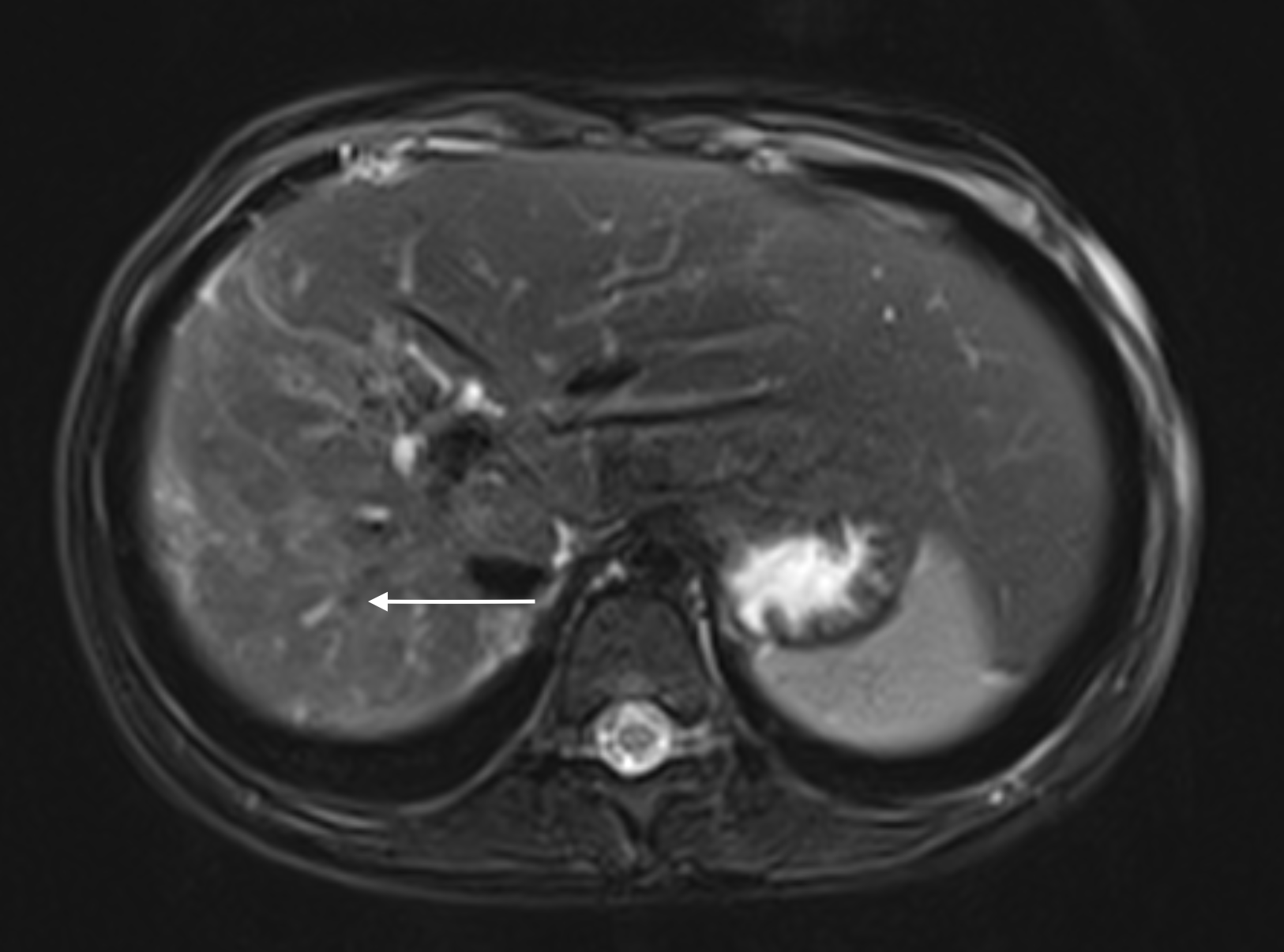

Supplement: Supplementary Figure 1 — MRI findings Axial T2 (A) MRI of the abdomen demonstrates that the right liver lobe was shrunken with a large patchy hyperintense signal, and coronal T1 (B) contrast enhanced MRI of the abdomen demonstrates delayed inhomogeneous intensified of the right liver lobe after enhancement. A hypointense signal was observed behind the of pancreatic head, with circular inhomogeneous enhancement. [file Image_1.tif]

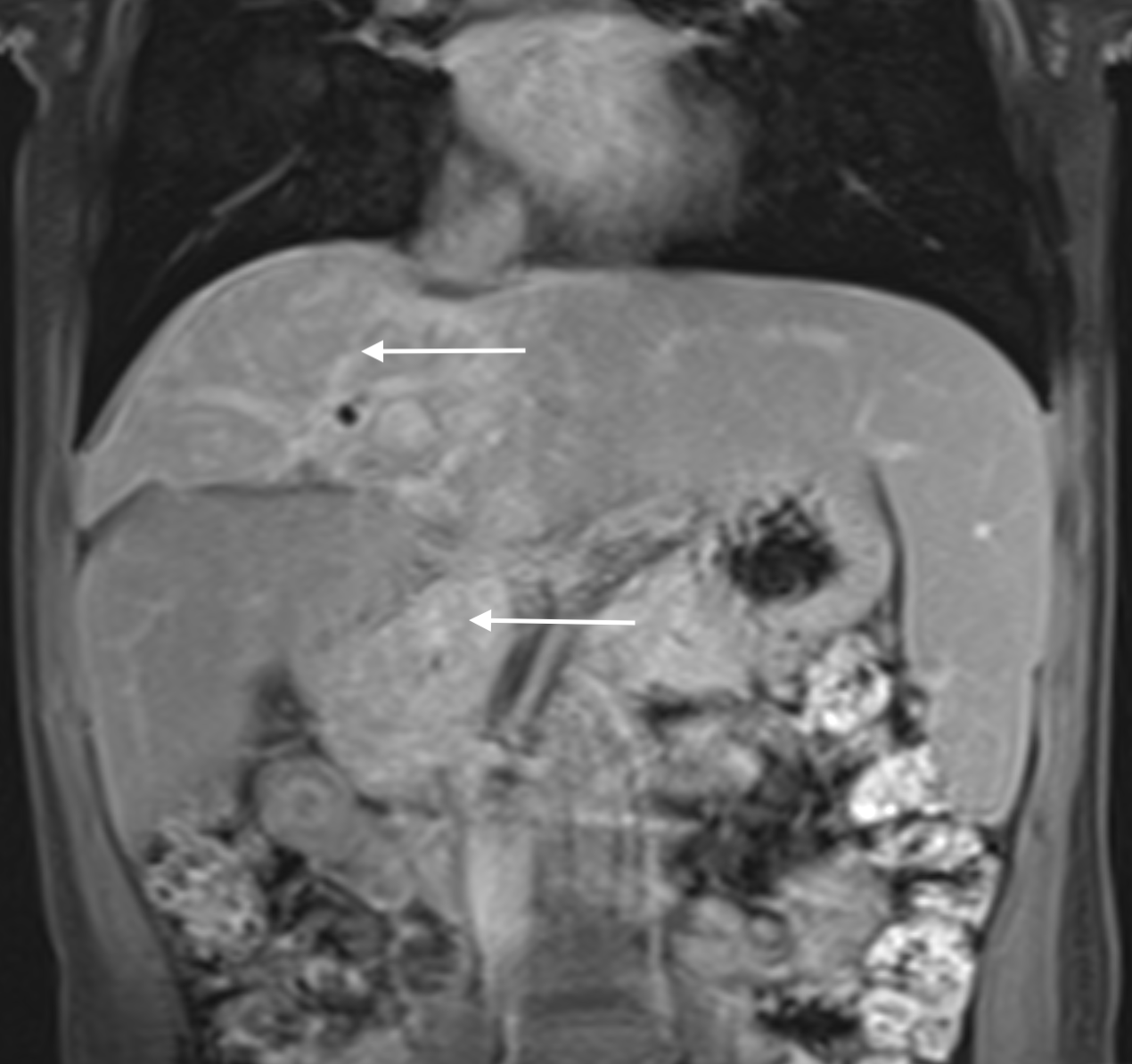

Supplement: Supplementary file 2 [file Image_2.tif]
